# Impact of Fungicide Mancozeb at Different Application Rates on Soil Microbial Populations, Soil Biological Processes, and Enzyme Activities in Soil

**DOI:** 10.1155/2014/702909

**Published:** 2014-11-16

**Authors:** Abhishek Walia, Preeti Mehta, Shiwani Guleria, Anjali Chauhan, C. K. Shirkot

**Affiliations:** ^1^Department of Basic Sciences (Microbiology Section), Dr. Yashwant Singh Parmar University of Horticulture and Forestry, Nauni, Solan 173230, India; ^2^DBT-IOC Centre for Bioenergy Research, Indian Oil R&D Centre, Sector-13, Faridabad, Haryana 121007, India; ^3^Department of Basic Sciences, Dr. Yashwant Singh Parmar University of Horticulture and Forestry, Nauni, Solan 173230, India

## Abstract

The use of fungicides is the continuous exercise particularly in orchard crops where fungal diseases, such as white root rot, have the potential to destroy horticultural crops rendering them unsaleable. In view of above problem, the present study examines the effect of different concentrations of mancozeb (0–2000 ppm) at different incubation periods for their harmful side effects on various microbiological processes, soil microflora, and soil enzymes in alluvial soil (pH 6.8) collected from apple orchards of Shimla in Himachal Pradesh (India). Low concentrations of mancozeb were found to be deleterious towards fungal and actinomycetes population while higher concentrations (1000 and 2000 ppm) were found to be detrimental to soil bacteria. Mancozeb impaired the process of ammonification and nitrification. Similar results were observed for nitrifying and ammonifying bacteria. Phosphorus solubilization was increased by higher concentration of mancozeb, that is, 250 ppm and above. In unamended soil, microbial biomass carbon and carbon mineralization were adversely affected by mancozeb. Soil enzymes, that is, amylase, invertase, and phosphatase showed adverse and disruptive effect when mancozeb used was above 10 ppm in unamended soil. These results conclude that, to lessen the harmful effects in soil biological processes caused by this fungicide, addition of higher amount of nitrogen based fertilizers is required.

## 1. Introduction

Agriculture relies on chemicals to control weeds, pest, and diseases. Pesticides include chemicals differing in their mode of action and are used by man as intentional addition to his environmental to control or eradicate a specific pest of economically important crops. Although, they are intended for harmful organisms but they also get into the nontarget system and bring about substantial impact on the ecosystem ranging from general poisoning to carcinogenic effects [1]. Microorganism is present in all environmental situations in which pesticides are used and they will, therefore, encounter pesticides, and however, inadvertently and probably react with them in some way. There is, therefore, a close relationship between pesticides and microorganisms because (i) these chemicals may have a deleterious effect on nontarget organisms and (ii) most of these pesticides being organic in nature could be metabolized by microorganisms resulting in modification of their activity. Pesticides gain entry into soil by a variety of means and their effect on soil microbial population may lead to elimination, decrease, or modification of microbial transformations and soil enzymes considered essential for soil fertility [2].

In Himachal Pradesh (HP), apple (*Malus domestica* Borkh), a major temperate horticulture fruit crop, receives maximum pesticides especially fungicides to combat diseases such as apple scab, powdery mildew, collar rot, stem cankers, and white root rot. However, significant yield losses in apple production due to apple scab, caused by the fungus* Venturia inaequalis*, are a major concern. The fungicide Mancozeb, in addition to other pesticides, is extensively used in apple orchards for control of apple scab, but it has the potential to affect the quality of soil, water, and air, with attendant risk to humans, flora, and fauna, mainly due to its persistence in soil [3].

Because of their relationship to soil biology and rapid response to changes in soil management, soil enzymes are recognized as sensitive indication of soil health and quality [4]. In fact, they have been related to soil physicochemical characters, microbial community structure [5], and disturbance [6]. The study of the secondary effects of fungicides on soil microbial activities and consequent impact on soil microbial activities and consequent impact on soil fertility is, therefore, of prime importance. Practically, little information is available on the effect of mancozeb on soil microbial activities, biological processes, and soil enzymes in HP.

## 2. Materials and Methods

### 2.1. Type and Preparation of Soil or Soil Sample

The soil used for incubation experiments was collected from a cropped field of apple orchard from Chopal area of district Shimla of HP. The soil is alluvial, typical of northern origin. Twenty samples of one kilogram soil each collected randomly were pooled together to make a composite sample. Soil samples were collected from 0 to 15 cm depth, air dried, and ground to pass a 2 mm sieve and stored in a humid atmosphere (80 relative humidity) at 20°C for maximum of seven days.

### 2.2. Physical and Chemical Analysis of the Soil

The characteristics of the soils are summarized in Table 1. Freshly procured samples of the soil were analyzed for the physical and chemical characteristics. Soil texture was determined by the hydrometer method [7], pH was determined after shaking the soil with deionized water (1 : 2.5 mass ratio) for 10 min, and organic matter was determined by rapid titration method [8]. Cation exchange capacity, available phosphorous, total-nitrogen, nitrate-nitrogen, and ammoniacal-nitrogen were determined by the procedures outlined by Jackson [9].

### 2.3. Effect of Mancozeb on Microbial Population

#### 2.3.1. Enumeration of Bacteria, Fungi, and Actinomycetes

Enumeration was done by using a standard dilution technique in which three portions (each of 10 gram fresh weight) were shaken separately in 90 mL sterilized distilled water in 250 mL flasks and further 10-fold dilutions were made for each sample. Bacteria, fungi, actinomycetes, and ammonifying and nitrifying bacteria were counted by plating 0.1 mL of suitable dilutions on separate plates and incubated at temperature 28°C. Soil extract agar, streptomycin rose bengal agar, and Kuster and Williams medium were the selective media for the enumeration of soil bacteria, fungi, and actinomycetes while ammonifying and nitrifying bacteria were enumerated using Bhuiya and Walker medium.

#### 2.3.2. Nitrification

Nitrite nitrogen and nitrate nitrogen were extracted by the method given by Jackson [9] and estimated by the method of Onken and Sunderman [10].

#### 2.3.3. Ammonification

Hundred grams of soil was mixed with sufficient peptone to give a nitrogen content of 500 ppm. Mancozeb was added as described earlier and incubated at 28°C. Appropriate soil samples were removed at 0, 1, 2, 3, and 4 weeks intervals and ammoniacal nitrogen was extracted [9] and estimated by the method of Onken and Sunderman [10].

#### 2.3.4. Phosphorous Solubilization

Phosphorous solubilization in soil was studied by incubating 100 g of soil with tricalcium phosphate to provide 200 ppm of phosphorous. Soil contained 0, 10, 100, 250, 500, 1000, and 2000 ppm of mancozeb and was incubated at 28°C. Soil samples were removed at 0, 1, 2, 3, and 4 weeks of intervals. Available phosphorous was extracted with alkaline sodium bicarbonate solution (pH 8.5) and estimated calorimetrically [9].

#### 2.3.5. Soil Microbial Biomass

Microbial biomass carbon was estimated by Chloroform-fumigation incubation technique [11].

#### 2.3.6. Carbon Mineralization

Carbon mineralization was determined by estimating the evolution of CO_2_ by the method of [12].

### 2.4. Enzymatic Determination

#### 2.4.1. Amylase [13]

Two grams of air dried soil samples was taken in a test tube and 0.3 mL of toluene was added. The mixture was shaken and allowed to stand for 15 minutes before the addition of buffer and substrate. 5 mL of 0.1 M sodium acetate buffer (pH 5.0) having 50 mg soluble starch was added to the reaction mixture. Reaction mixture was incubated for 24 h at 28°C. After incubation, 10 mL of distilled water was added and soil suspension was centrifuged at 12,000 ×g for 10 minutes. Supernatant was taken and analyzed for reducing sugars by the method of Nelson [14].

#### 2.4.2. Invertase [13]

Invertase activity was assayed in a manner identical to that of amylase, except that buffer used was sodium acetate (0.1 M, pH 5.5) containing 18 mM sucrose and incubation period was 3 h.

#### 2.4.3. Phosphatase [15]

One gram soil taken in a test tube was incubated with 1 mL of 5 mM buffered sodium-p-nitrophenyl phosphate in acetate buffer (pH 5.2) and 0.3 mL toluene at 37°C for 1 h. Determination of p-nitrophenol involved the calorimetric analysis of the extract obtained by treating the incubated soil sample with 4 mL water, 10 mL of 0.5 M CaCl_2_, and 4 mL of 0.5 M sodium hydroxide and by filtering (Whatman no. 42) the suspension obtained by shaking the mixture for one minute and the absorbance of yellow color of p-nitrophenol released was determined spectrophotometrically at 420 nm.

## 3. Results

### 3.1. Effect of Mancozeb on Soil Biological Processes

#### 3.1.1. Effect of Mancozeb on Soil Microflora

The results showed gradual increase in bacterial population with increase in mancozeb concentration up to 250 ppm at all the incubation period studied. Bacterial population at higher concentrations (1000 and 2000 ppm) decreased significantly. Fungal population was not affected by the presence of 10 ppm mancozeb in the soil. Increase in mancozeb concentrations up to 100 ppm and above decreased fungal population. Mancozeb generally decreased actinomycetes population at all the concentrations studied and, at 1000 and 2000 ppm, it adversely affected the actinomycetes population. With increase in incubation period, actinomycetes population decreased while the fungal population increased slightly. The bacterial population on an average regained the original level after four weeks of incubation (Data not shown).

#### 3.1.2. Effect of Mancozeb on Nitrification

**Figure 1:**
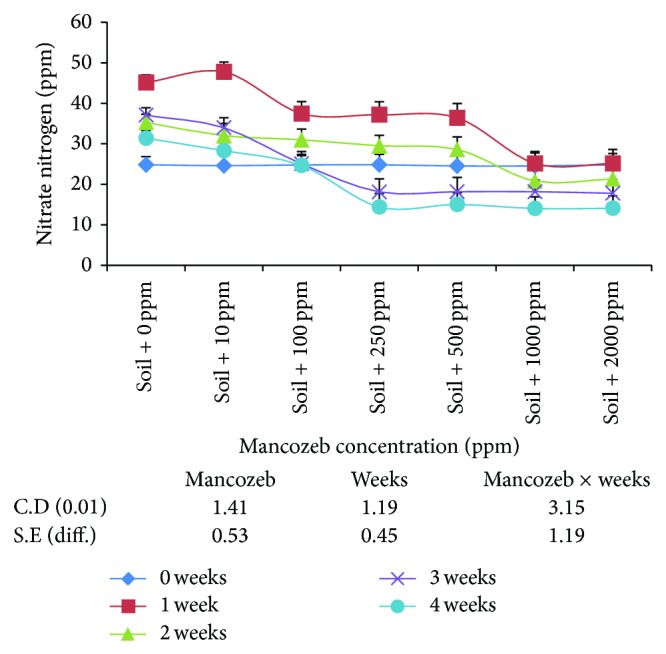
Effect of mancozeb on nitrification of 100 ppm of diammonium hydrogen phosphate applied to soil.

The results on the effect of varying concentrations of mancozeb on nitrification of diammonium hydrogen phosphate in soil (Figure 1) revealed that average nitrate-nitrogen (NO_3_^−^–N) decreased significantly with the increase in mancozeb concentrations from 0 ppm (34.74 ppm NO_3_^−^–N) to 2000 ppm (20.63 ppm NO_3_^−^–N). However, the difference in NO_3_^−^–N between 250 and 500 ppm and 1000 and 2000 ppm was nonsignificant amongst themselves. The effect of incubation period revealed significant increase in average NO_3_^−^–N after one week of incubation. Further increase in incubation period had inhibitory effect on nitrification, as average NO_3_^−^–N decreased to 20.26 ppm at the end of four weeks of incubation.

The interaction between mancozeb concentration and incubation period was found to be statistically significant. Incubation of soil samples for one week resulted in significant increase in NO_3_^−^–N at 0, 10, 100, 250, and 500 ppm mancozeb concentrations. At the end of second week of incubation period, the presence of mancozeb up to 100 ppm decreased NO_3_^−^–N from 35.28 ppm (0 ppm) to 31.01 ppm. Increase in mancozeb concentration from 500 ppm to 1000 ppm significantly decreased NO_3_^−^–N to minimum 20.80 ppm. At the end of three weeks of incubation period, NO_3_^−^–N increased significantly in the presence of 10 and 100 ppm mancozeb. However, difference in the decrease in NO_3_^−^–N at 250, 500, 1000, and 2000 ppm was nonsignificant amongst themselves. Similar trend was observed at the end of four weeks of incubation period. Thus, mancozeb at 250 ppm strongly inhibited nitrification after three weeks of incubation and the inhibitory effect was not overcome even after four weeks.

#### 3.1.3. Effect of Mancozeb on Nitrifying Bacteria

**Figure 2:**
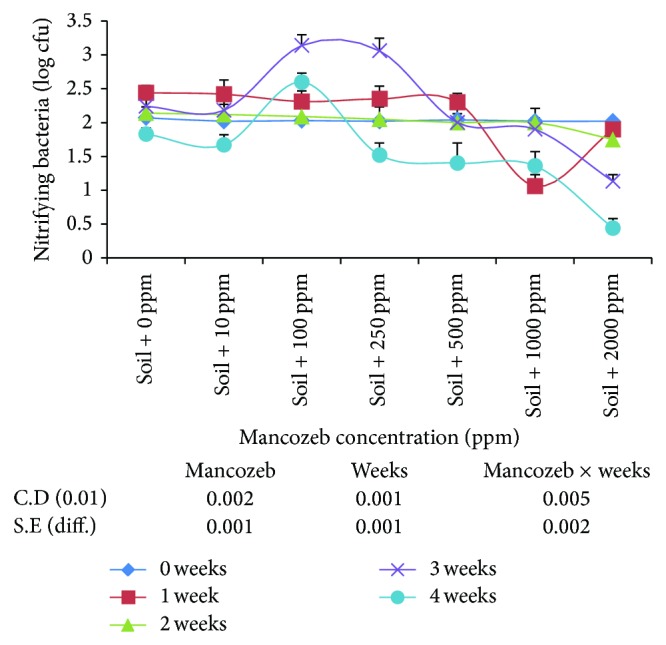
Effect of mancozeb on nitrifying bacterial population.

The results on varying concentrations of mancozeb (0–2000 ppm) on nitrifying bacteria (Figure 2) showed gradual decrease in average number of nitrifying bacterial population from 2.14 log cfu at zero ppm to 1.44 log cfu at 2000 ppm. Increase in incubation period had inhibitory effect on nitrifying bacterial population as it decreased gradually with increase in incubation period.

Interaction studied between mancozeb concentration and incubation period was found to be statistically significant. On day zero, the difference in nitrifying bacterial population was nonsignificant at 1000 and 2000 ppm mancozeb concentrations. At the end of one-week incubation period, the population increased considerably at 0, 10, 100, 250, and 500 ppm mancozeb. However, nitrifying bacterial population decreased significantly at the end of second week of incubation. At the end of the third and fourth week of incubation period, nitrifying population decreased considerably at higher mancozeb concentrations (1000 and 2000 ppm).

#### 3.1.4. Effect of Mancozeb on Ammonification

**Figure 3:**
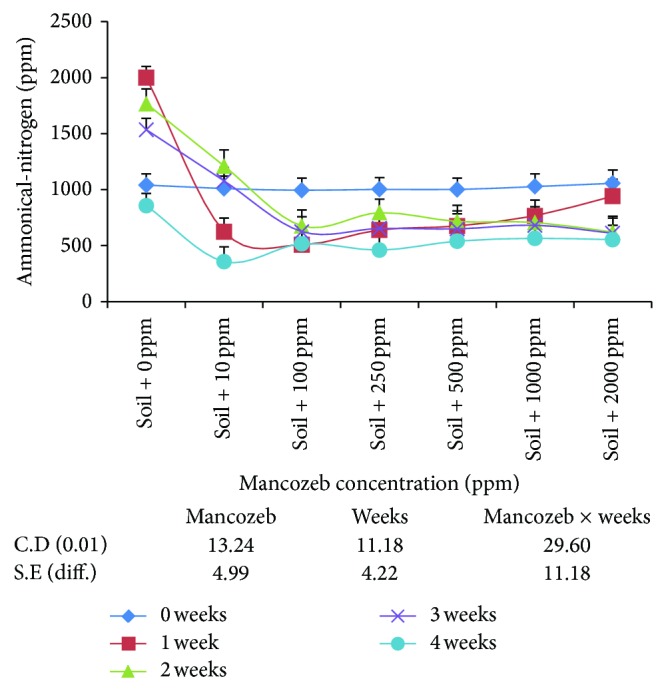
Effect of mancozeb on ammonification of 500 ppm of peptone applied to soil.

The effect of mancozeb (0–2000 ppm) on the production of ammoniacal-nitrogen (NH_4_^+^–N) from applied peptone was studied in apple orchard soil. The findings (Figure 3) showed significant decrease in the average NH_4_^+^–N in mancozeb treated soil as compared to control. The amount of average ammoniacal-nitrogen was at par amongst 250 and 500 ppm and 1000 and 2000 ppm of mancozeb concentrations. Average ammoniacal-nitrogen decreased with increase in incubation period irrespective of mancozeb concentrations.

The interaction between mancozeb concentration and incubation period was found to be statistically significant. Ammoniacal-nitrogen decreased in presence of different concentrations of mancozeb at all the incubation period except in the presence of 10 ppm mancozeb at the end of second and third week of incubation where NH_4_^+^–N increased significantly over the values obtained on day zero. Thus, mancozeb markedly decreased ammonification even at as low as 10 ppm in apple orchard soil.

#### 3.1.5. Effect of Mancozeb on Ammonifying Bacteria

**Figure 4:**
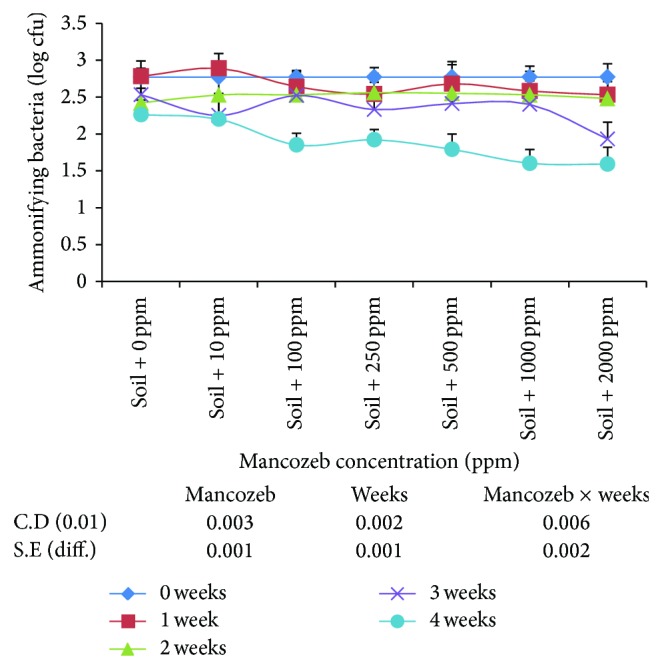
Effect of mancozeb on ammonifying bacterial population.

The results on the effect of mancozeb (0–2000 ppm) on ammonifying bacteria (Figure 4) indicated significant decrease in their average population at various concentrations. Incubation period studied also had significant effect on ammonifying bacteria. On an average, ammonifying bacteria decreased gradually with increase in incubation period.

The interaction studied between mancozeb concentration and incubation period was statistically significant. At the end of one-week incubation, there was decrease in number of ammonifying bacteria at mancozeb concentration above 10 ppm. Incubation of soil sample for second, third, and fourth weeks, however, decreased ammonifying bacteria at all the concentrations studied.

#### 3.1.6. Effect of Mancozeb on Phosphorous Solubilization

**Figure 5:**
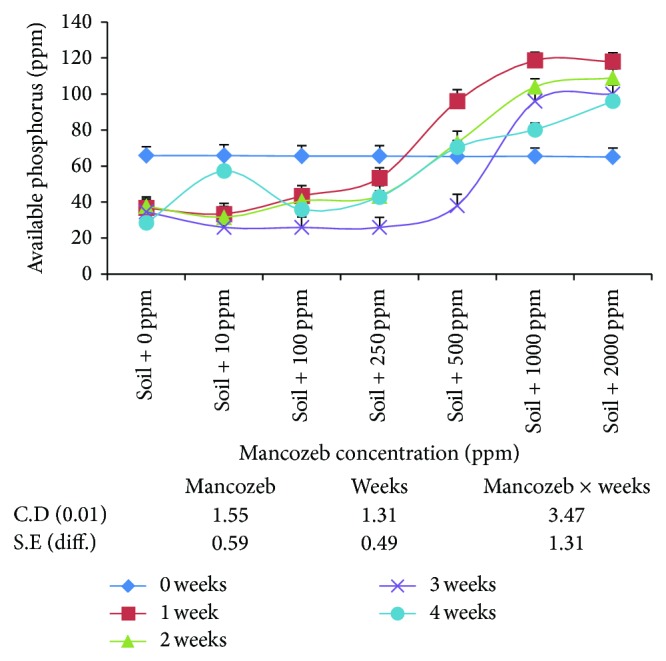
Effect of mancozeb on phosphorus solubilization of 200 ppm of tricalcium phosphate applied to soil nitrifying bacterial population.

From the results (Figure 5), it was observed that lower concentrations (0, 10, and 100 ppm) of mancozeb had little effect on the average amount of available phosphorous released. Above 100 ppm, the amount of phosphorous increased significantly with increase in mancozeb concentrations and maximum value was obtained at 2000 ppm mancozeb (97.60 ppm). The effect of incubation period revealed significant increase in average phosphorous after one week of incubation (71.34 ppm). Further incubation had inhibitory effect on phosphorous solubilization as average phosphorous decreased to 49.43 ppm at the end of three weeks.

The interaction between mancozeb concentration and incubation period was also found to be significant. The effect of various concentrations of mancozeb on phosphorous solubilization on day zero was nonsignificant. At the end of one-week-incubation period, phosphorous solubilization decreased significantly to 250 ppm. Above 250 ppm, it increased significantly with nonsignificant difference between 1000 and 2000 ppm of mancozeb concentrations. After incubating soil samples for two weeks, phosphorous solubilization decreased as compared to the value obtained at the end of first week. However, difference between 100 and 250 ppm mancozeb was nonsignificant. At the end of third week of incubation period, phosphorous solubilization further decreased significantly with nonsignificant difference between 10, 100, and 250 ppm mancozeb concentrations. At the end of four weeks of incubation, phosphorous solubilization increased significantly up to 500 ppm but decreased at 1000 and 2000 ppm as compared to amount of phosphorous obtained after three weeks of incubation.

#### 3.1.7. Effect of Mancozeb on Carbon Mineralization

**Figure 6:**
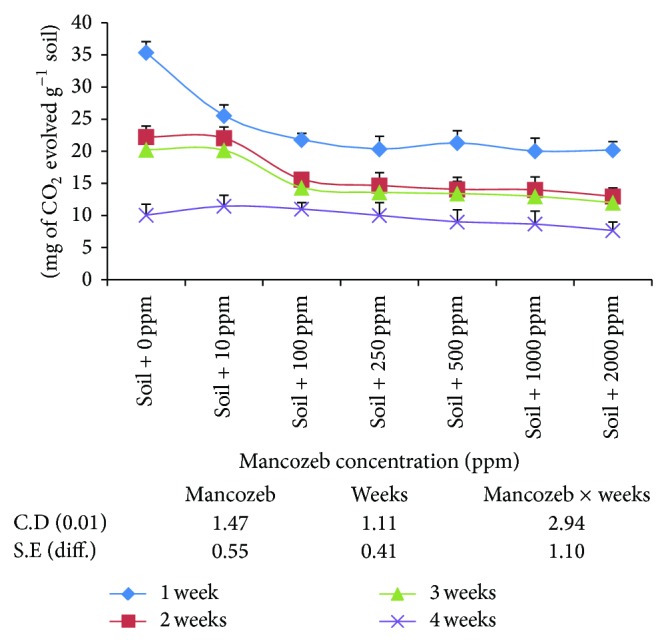
Effect of mancozeb on carbon-dioxide evolution.

The results on the effect of mancozeb on evolution of carbon dioxide (CO_2_) in soil (Figure 6) showed that average CO_2_-evolution decreased gradually with increase in mancozeb concentrations from 10 ppm (19.80 mg) to 2000 ppm (13.21 mg) with statistically nonsignificant difference amongst 250, 500, 1000, and 2000 ppm. The effect of incubation period revealed significant decrease in average CO_2_-production with increase in incubation period.

The interaction between incubation period and mancozeb concentration was also found to be significant. The amount of CO_2_-evolved decreased gradually with increase in mancozeb concentration irrespective of the incubation period.

#### 3.1.8. Effect of Mancozeb on Soil Microbial Biomass Carbon

**Figure 7:**
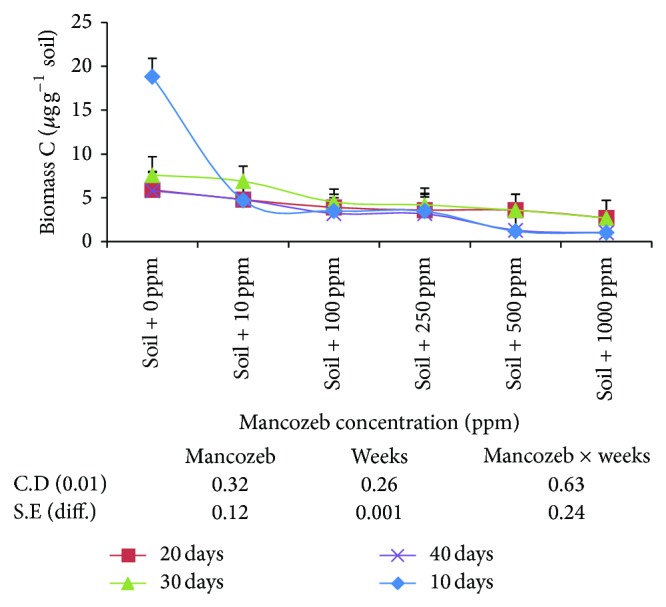
Effect of mancozeb on soil microbial biomass carbon.

Effect of mancozeb concentrations (0–1000 ppm) on soil microbial biomass was studied by measuring CO_2_–C production from soil. The results (Figure 7) revealed gradual decrease in average microbial biomass carbon with increase in mancozeb concentrations from 9.52 *µ*g/g at 0 ppm to 1.86 *µ*g/g at 1000 ppm. Incubation period also had significant effect on soil microbial biomass C and it decreased with increase in incubation period.

Interaction studied between mancozeb concentration and incubation period was found to be significant. After incubating soil sample for 10 days, maximum microbial biomass C was obtained in the absence of mancozeb (18.8 *µ*g/g) and lowest value was obtained at 1000 ppm (1.02 *µ*g/g). After incubating soil samples for 20 days, microbial biomass C decreased at 0 and 10 ppm and increase in microbial biomass C at 100, 250, and 500 ppm was statistically nonsignificant amongst themselves. Further incubation of soil sample for thirty days increased microbial biomass at all the concentrations with nonsignificant difference amongst 100 and 250 ppm. At the end of forty days, microbial biomass C decreased with nonsignificant difference amongst 100 and 250 ppm and 500 and 1000 ppm.

### 3.2. Effect of Mancozeb on Soil Enzyme

#### 3.2.1. Amylase

**Figure 8:**
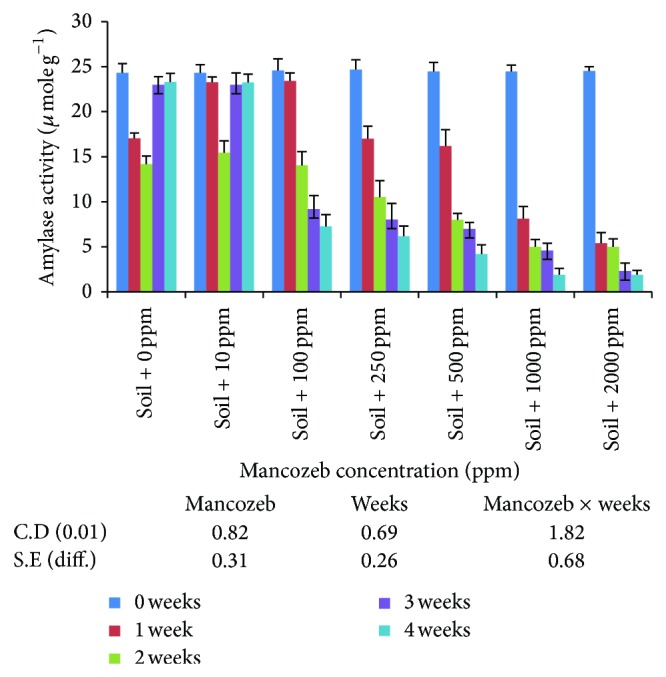
Effect of mancozeb on amylase in soil.

Amylase activity (Figure 8) at 10 ppm mancozeb concentration was significantly increased over the control. Increase in mancozeb concentration above 10 ppm decreased average amylase activity. Average amylase activity decreased significantly with increase in incubation period irrespective of mancozeb concentrations.

The interaction studied indicated significant relationship between mancozeb concentration and incubation period. Amylase activity on day zero was at par irrespective of the mancozeb concentrations. After one week of incubation, amylase activity decreased significantly at all the concentrations with nonsignificant difference amongst 10 and 100 ppm mancozeb concentrations. When soil was incubated for two weeks, amylase activity decreased significantly. At the end of three- and four-week incubation period, amylase activity at 0 and 10 ppm was restored to the original value obtained on day zero. However, the increase was statistically nonsignificant amongst them. Further increase in mancozeb concentrations significantly decreased amylase activity. Thus, mancozeb at 100 ppm and above adversely affected amylase activity and the inhibitory effect was not overcome even after four weeks of incubation.

#### 3.2.2. Invertase

**Figure 9:**
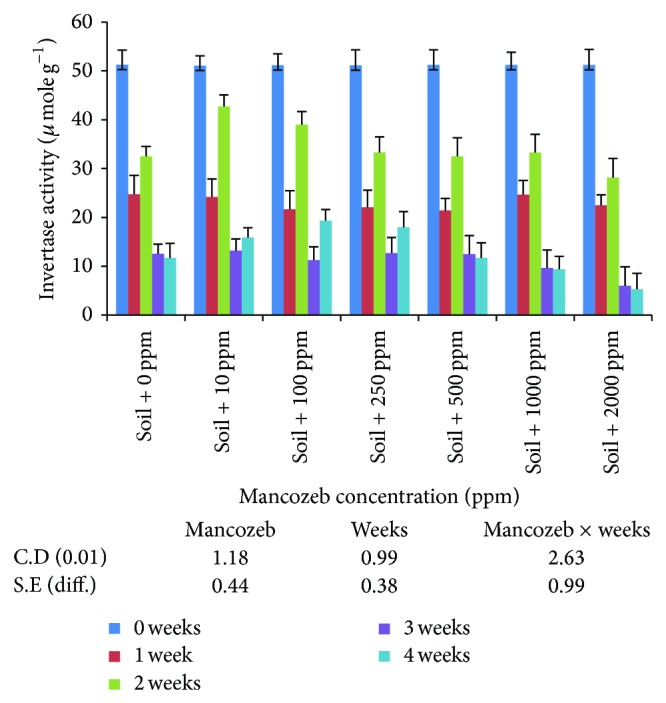
Effect of mancozeb on invertase in soil.

The results on invertase activity (Figure 9) showed increase in average invertase activity from 26.54 U to 29.40 U when mancozeb concentration was increased from 0 to 10 ppm. The average invertase activity was decreased gradually with increase in mancozeb concentrations above 10 ppm. However, the activity was at par amongst 100, 250 ppm and 500 and 1000 ppm of mancozeb. Incubation period had significant effect on average invertase activity. After one week of incubation, the invertase activity was reduced from 51.19 U (0 days) to 23.03 U (1 week). At the end of second week of incubation, invertase activity increased from 23.03 U (first week) to 34.50 U (second week). Continued incubation significantly decreased invertase activity.

Interaction studied between mancozeb concentration and incubation period showed significant difference in invertase activity at different intervals of time and mancozeb concentrations. On day zero, the invertase activity at various mancozeb concentrations was nonsignificant. At the end of one week of incubation, invertase activity decreased significantly over day zero at all the concentrations. The activity was statistically the same at 0 and 10 ppm; 250 and 500 ppm; and 1000 and 2000 ppm. At the end of two-week incubation period, invertase activity increased at all the mancozeb concentrations with maximum activity (42.70 U) in presence of 10 ppm mancozeb. It was observed that, at the end of three and four weeks of incubation period, invertase activity decreased significantly with statistically nonsignificant difference between various mancozeb concentrations.

#### 3.2.3. Phosphatase

**Figure 10:**
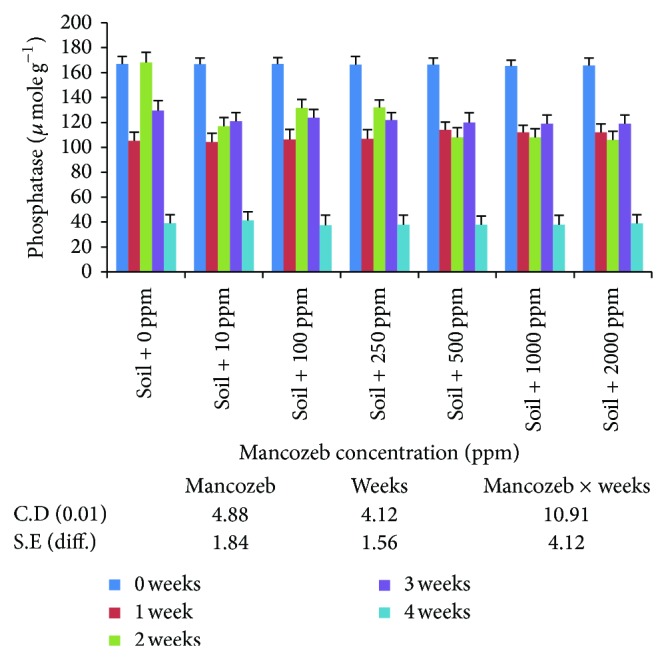
Effect of mancozeb on phosphatase in soil.

The results on phosphatase activity (Figure 10) indicated maximum average phosphatase activity at zero ppm (121.8 U). Average phosphatase activity decreased significantly to 113.2 U at 100 ppm mancozeb concentration. The increase in mancozeb concentrations above 100 ppm decreased phosphatase activity with nonsignificant difference between various mancozeb concentrations used. With the increase in incubation period, significant decrease in average phosphatase activity was observed. Minimum average phosphatase activity (38.7 U) was obtained after incubating soil sample for four weeks.

Interaction studied indicated a nonsignificant difference in enzyme activity on day zero. At the end of one-week incubation period, the activity was significantly decreased and varied between 105.3 U (0 ppm) to 114.0 U (500 ppm). The difference in enzyme activity between various mancozeb concentrations was nonsignificant. Increase in incubation period for two weeks resulted in increase in enzyme activity. The activity at 0 ppm (168.2 U) decreased to 117.0 U at 10 ppm but increased to 131.7 U at 100 ppm and 132.1 U at 250 ppm mancozeb concentrations. Further increase in mancozeb concentrations did not affect the activity as the difference was statistically nonsignificant. Continued incubation for four weeks decreased enzyme activity at all the mancozeb concentrations.

## 4. Discussion

When any pesticide is applied to control harmful microorganisms, it invariably affects the abundance and performance of other microorganisms. In the present study, it has been observed that mancozeb increased bacterial population up to 250 ppm at all the time intervals but higher concentrations (1000 and 2000 ppm) had adverse effect. Fungal and actinomycetes populations decreased in mancozeb treated soil at concentrations above 10 ppm and degree of reduction is related to the amount of fungicide applied initially. This effect on soil microflora may be either due to the effect of mancozeb or due to the toxic degradation products like carbon disulphide. Similar disruptive effect on soil microflora by dithiocarbamates [16], mancozeb [17], and other fungicides [18, 19] has also been reported earlier in the nonrhizosphere soil. The increase in bacterial population might be due to decrease in competition for existence and nutrients with fungi and actinomycetes which are inhibited by mancozeb [20].

In the present study, mancozeb is found to decrease nitrification (Figure 1) at all the concentrations studied which is well agreed with earlier reports [21]. Concentrations of mancozeb above 10 ppm may have inhibitory effect on the specialized group of nitrifying bacteria and inhibitory effect was constant beyond 250 ppm to 500 ppm. This is supported by the results (Figure 2), where nitrifying bacteria are adversely affected by the concentrations of mancozeb above 10 ppm. The effect of mancozeb concentrations up to 100 ppm in soil is removed by the end of four weeks of incubation as NO_3_^−^–N is equal to that on day zero. This may be due to the degradation of lower concentration of mancozeb by soil microflora to the level which is not inhibitory to nitrifying bacteria. Higher concentration seems to be persistent in its toxicity towards microorganisms involved in nitrification. The effect of lower concentrations of mancozeb on nitrification is in agreement with the earlier reports [22] in the soil with entirely different physical and chemical characteristics.

The quantity of NH_4_^+^–N in mancozeb treated soil is found to decrease considerably as compared to the control even at lower rates of its application (Figure 3). The magnitude of decrease has been related to the amount of fungicide applied initially as minimum NH_4_^+^–N is obtained at highest mancozeb concentration (2000 ppm) which is agreed to the reports shown earlier by [23, 24]. A higher mancozeb concentration seems to be persistent in its toxicity towards ammonifiers. Dubey and Rodriguez [25] reported nonpersistent effect of ethylene-bis-dithiocarbamate on ammonification in acid laterite, clay, and alluvial soil. The significant decrease in the population of ammonifying bacteria is in agreement with the earlier results [17] which observed decrease in aerobic nitrogen cycle population which adversely affected nitrogen mineralization.

The present study reveals that the process of phosphorus solubilization is not disturbed by mancozeb treatment as high as 250 ppm but is found to increase at higher rates of its application. Ahemad and Khan [26, 27] similarly showed that P-solubilization was in a minor way affected at recommended doses but majorly affected at higher doses of fungicides. It may be because of the reason that soil microorganisms which can solubilize added insoluble phosphates are enriched in presence of higher mancozeb concentrations. In addition, the number of total microbial population is decreased and it may result in the lesser utilization of the released phosphorus.

The effect of mancozeb on evolution of carbon-dioxide (CO_2_) in unamended soil (Figure 6) showed that average CO_2_-evolution decreased gradually with increase in mancozeb concentrations from 10 ppm (19.80 mg) to 2000 ppm (13.21 mg) and coincides with the results of Cernohlavkova et al. 2009 [28] which showed that carbon mineralization was affected with higher doses of fungicides. The slight increase in CO_2_-evolution from soil amended with leaf litter over nonamended soil (data not shown) might be partly due to decomposition of leaf litter resulting in the evolution of CO_2_ and due to selective increase in the population of some fungal species in treated leaf litter. The present results are in agreement with those obtained with mancozeb where 5 to 100 times decrease in population of carbon-mineralizing microorganisms at 10 ppm concentration have been reported [17].

In the present study, the amount of microbial biomass C in the unamended soil (Figure 7) was less as compared to the biomass C in apple leaf litter amended soil (data not shown). Presumably, since no substrate in the form of apple leaf litter was added into the soil, biomass must have utilized soil organic matter as its principle energy source [29]. Smith et al. [30] also showed that benomyl fungicide has no effect on soil microbial biomass C only due to the addition of organic matter.

In the present study, the differences in enzyme activities indicate that effect of mancozeb on soil enzymes is variable [18]. However, the enzyme activities are invariably present and enzymes are not rendered completely inactive. This may be due to the fact that enzyme may become established in the soil due to the formation of clay-enzyme complex [31]. The decrease in enzyme activity may be due to decrease in the microbial population, destruction, or inactivation of preexisting soil enzymes and substrate limiting for enzyme induction. These results were also supported by [19, 32] who found that invertase enzyme activity decreased with increase in fungicide concentration. Sukul [2] also reported that the activity of soil enzymes was considerably affected by the time of fungicide action.

Lower concentrations of mancozeb increase soil phosphatase activity. This can be due to the presence of zinc and manganese ions in mancozeb which might enhance the enzyme activity. Dick and Tabatabai [33] also found that zinc and manganese at low concentrations activated soil phosphatase activity, presumably by formation of a substrate-metal-enzyme bridge. Same results were also found in case of amylase and invertase where the enzymatic activities increased to some extent at low concentration with increase in incubation time. These results were also supported by Tu [1] where captafol and chlorothalonil suppressed invertase activity for one day temporarily in a sandy loam soil and later on, after 2 days, the inhibitory effect diminished.

## 5. Conclusion

In conclusion, the fungicide mancozeb has a considerable deleterious impact on soil microflora, nitrification, ammonification, soil microbial biomass, carbon mineralization, and soil enzymes which may result in harmful effects on nutrient uptake and plant growth. These findings suggest that the use of mancozeb to control plant diseases in apple orchard soil requires simultaneous application of large quantities of nitrogen based fertilizers.

## Figures and Tables

**Table 1:** Characteristics of the soil used in the incubation studies.

Characteristics	Average value
Water holding capacity	37.5%
Cation exchange	11 meq 100 g^−1^
Soil texture:	
(i) Sand	52%
(ii) Silt	20%
(iii) clay	28%
pH	6.8
Nitrogen	
(i) Ammoniacal nitrogen	21.4 ppm
(ii) Nitrate-N	5.2 ppm
(iii) Nitrite-N	N.D^*^
(iv) Total nitrogen	0.06%
Organic carbon	1.45%
Phosphate-P	32.2 ppm

^*^N.D.: Not determined.

## References

[1] Tu C. M. (1993). Effect of fungicides, captafol and chlorothalonil, on microbial and enzymatic activities in mineral soil. *Journal of Environmental Science and Health B*.

[2] Sukul P. (2006). Enzymatic activities and microbial biomass in soil as influenced by metalaxyl residues. *Soil Biology & Biochemistry*.

[3] Huber S., Syed B., Freudenschuss A., Ernstsen V., Loveland P. (2001). Proposal for a European soil monitoring and assessment framework.

[4] Caldwell B. A. (2005). Enzyme activities as a component of soil biodiversity: a review. *Pedobiologia*.

[5] Kourtev P. S., Ehrenfeld J. G., Haggblom M. (2002). Exotic plant species alter the microbial community structure and function in the soil. *Ecology*.

[6] Boerner R. E. J., Decker K. L. M., Sutherland E. K. (2000). Prescribed burning effects on soil enzyme activity in a southern Ohio hardwood forest: a landscape-scale analysis. *Soil Biology & Biochemistry*.

[7] Boyoucous C. J. (1936). Directions for making mechanical analysis of soil by the hydrometer method. *Soil Science*.

[8] Walkley A., Black I. A. (1934). An examination of the Degtjareff method for determining soil organic matter and a proposed modification of the chrom ic acid titration method. *Soil Science*.

[9] Jackson M. L. (1967). *Soil Chemical Analysis*.

[10] Onken A. B., Sunderman H. D. (1977). Colorimetric determination of exchangeable ammonium, nitrate and nitrite in a single soil extract. *Agronomy Journal*.

[11] Jenkinson D. S., Powlson D. S. (1976). The effects of biocidal treatments on metabolism in soil—V: a method for measuring soil biomass. *Soil Biology and Biochemistry*.

[12] Pramer C., Schmidt A., Black C. A. (1964). Organic matter. *Methods of Soil Analysis*.

[13] Cole M. A. (1977). Lead inhibition of enzyme synthesis in soil. *Applied and Environmental Microbiology*.

[14] Nelson N. (1944). A photometric adaptation of the Somogyi method for the determination of glucose. *The Journal of Biological Chemistry*.

[15] Tabatabai M. A., Bremner J. M. (1969). Use of p-nitrophenyl phosphate for assay of soil phosphatase activity. *Soil Biology and Biochemistry*.

[16] Agnihotri V. P. (1974). Thiram induced changes in soil microflora, their physiological activity and control of damping off in chillies (*Capsicum annum*). *Indian Journal of Experimental Biology*.

[17] Doneche B., Seguin G., Ribereau-Gayon P. (1983). Mancozeb effect on soil microorganisms and its degradation in soils.. *Soil Science*.

[18] Shan M., Fang H., Wang X., Feng B., Chu X. Q., Yu Y. L. (2006). Effect of chlorpyrifos on soil microbial populations and enzyme activities. *Journal of Environmental Sciences*.

[19] Fawole O. B., Aliko M., Olowonihi T. E. (2010). Effects of a Carbendazim-Mancozeb fungicidal mixture on soil microbial populations and some enzyme activities in soil. *Agrosearch*.

[20] Anderson J. R., Hill I. R., Wright S. L. J. (1978). Pesticide effects on non-target soil microorganisms. *Pesticide Microbiology*.

[21] Chen S.-K., Edwards C. A., Subler S. (2001). Effects of the fungicides benomyl, captan and chlorothalonil on soil microbial activity and nitrogen dynamics in laboratory incubations. *Soil Biology and Biochemistry*.

[22] Chen S.-K., Edwards C. A. (2001). A microcosm approach to assess the effects of fungicides on soil ecological processes and plant growth: comparisons of two soil types. *Soil Biology and Biochemistry*.

[23] Das A. C., Mukherjee D. (2000). Influence of insecticides on microbial transformation of nitrogen and phosphorus in typic Orchragualf soil. *Journal of Agricultural and Food Chemistry*.

[24] Demanou J., Monkiéjé A., Njin T., Foto S. M., Nola M., Togouet S. H. Z., Kemka N. (2004). Changes in soil chemical properties and microbial activities in response to the fungicide Ridomil Gold plus copper. *International Journal of Environmental Research and Public Health*.

[25] Dubey H. D., Rodriguez R. L. (1970). Effect of dyrene and maneb on nitrification and ammonification, and their degradation in tropical soils. *Soil Science Society of America Journal*.

[26] Ahemad M., Khan M. S. (2010). Influence of selective herbicides on plant growth promoting traits of phosphate solubilizing Enterobacter asburiae strain PS2. *Research Journal of Microbiology*.

[27] Ahemad M., Khan M. S. (2012). Effect of fungicides on plant growth promoting activities of phosphate solubilizing *Pseudomonas putida* isolated from mustard (*Brassica compestris*) rhizosphere. *Chemosphere*.

[28] Černohlávková J., Jarkovský J., Hofman J. (2009). Effects of fungicides mancozeb and dinocap on carbon and nitrogen mineralization in soils. *Ecotoxicology and Environmental Safety*.

[29] Joergensen R. G., Brookes P. C., Jenkinson D. S. (1990). Survival of the soil microbial biomass at elevated temperatures. *Soil Biology and Biochemistry*.

[30] Smith M. D., Hartnett D. C., Rice C. W. (2000). Effects of long-term fungicide applications on microbial properties in tallgrass prairie soil. *Soil Biology & Biochemistry*.

[31] Dick R. P., Tabatabai M. A. (1987). Factors affecting hydrolysis of polyphosphates in soils. *Soil Science*.

[32] Srinivasulu M., Rangaswamy V. (2006). Activities of invertase and cellulase as influenced by the application of tridemorph and captan to groundnut (*Arachis hypogaea*) soil. *African Journal of Biotechnology*.

[33] Dick W. A., Tabatabai M., Metting F. B. (1993). Significance and potential uses of soil enzymes. *Soil Microbial Ecology: Applications in Agricultural and Environmental Management*.

